# Engineering and Characterization of a Fluorescent Native-Like HIV-1 Envelope Glycoprotein Trimer

**DOI:** 10.3390/biom5042919

**Published:** 2015-10-23

**Authors:** Kwinten Sliepen, Thijs van Montfort, Gabriel Ozorowski, Laura K. Pritchard, Max Crispin, Andrew B. Ward, Rogier W. Sanders

**Affiliations:** 1 Department of Medical Microbiology, Academic Medical Center, University of Amsterdam, Amsterdam 1105 AZ, The Netherlands; E-Mails: k.h.sliepen@amc.uva.nl (K.S.); t.vanmontfort@amc.uva.nl (T.M.); 2 Department of Integrative Structural and Computational Biology, IAVI Neutralizing Antibody Center, Collaboration for AIDS Vaccine Discovery (CAVD), Center for HIV/AIDS Vaccine Immunology and Immunogen Discovery, The Scripps Research Institute, La Jolla, CA 92037, USA; E-Mails: gozorows@scripps.edu (G.O.); abward@scripps.edu (A.B.W.); 3 Department of Biochemistry, Oxford Glycobiology Institute, University of Oxford, South Parks Road, Oxford OX1 3QU, UK; E-Mails: laura.pritchard@linacre.ox.ac.uk (L.K.P.); max.crispin@bioch.ox.ac.uk (M.C.); 4 Department of Microbiology and Immunology, Weill Medical College of Cornell University, New York, NY 10065, USA

**Keywords:** green fluorescent protein (GFP), HIV-1 Env, SOSIP, superfolder GFP, protein engineering

## Abstract

Generation of a stable, soluble mimic of the HIV-1 envelope glycoprotein (Env) trimer on the virion surface has been considered an important first step for developing a successful HIV-1 vaccine. Recently, a soluble native-like Env trimer (BG505 SOSIP.664) has been described. This protein has facilitated major advances in the HIV-1 vaccine field, since it was the first Env immunogen that induced consistent neutralizing antibodies against a neutralization-resistant (tier 2) virus. Moreover, BG505 SOSIP.664 enabled elucidation of the atomic resolution structure of the Env trimer and facilitated the isolation and characterization of new broadly neutralizing antibodies against HIV-1. Here, we designed and characterized the BG505 SOSIP.664 trimer fused to fluorescent superfolder GFP (sfGFP), a GFP variant that allows efficient folding (BG505 SOSIP.664-sfGFP). Despite the presence of the sfGFP, the Env protein largely retained its morphology, antigenicity, glycan composition, and thermostability. In addition, we show that BG505 SOSIP.664-sfGFP can be used for fluorescence-based assays, such as flow cytometry.

## 1. Introduction

The acquired immune deficiency syndrome (AIDS) caused by the human immunodeficiency virus (HIV-1) is one of the leading deadly communicable diseases and an effective vaccine is elusive. A protective vaccine against HIV-1 should probably be capable of inducing broadly neutralizing antibodies (bNAbs) in order to prevent HIV-1 acquisition [1,2]. Over the last five years, many bNAbs have been isolated from HIV-1 infected individuals that target the HIV-1 envelope glycoprotein (Env) trimer and neutralize viruses from different subtypes, providing evidence that such antibodies can be induced. However, in contrast to natural infection, no Env-based vaccine has been able to induce bNAbs, one reason could be because these vaccines do not recapitulate the native Env trimer.

The Env trimer consists of three non-covalently linked gp120 and gp41 heterodimers that derive from a furin cleavable gp160 precursor protein. Env proteins are typically truncated before the transmembrane domain of gp41 to create soluble Env immunogens. However, unmodified gp140 proteins are unstable and do not trimerize efficiently [3]. A common technique to “stabilize” gp140 has been to remove the cleavage site between the gp120 and the gp140 ectodomain (gp41_ECTO_), with or without further addition of a heterologous trimerization domain [4]. The resulting uncleaved gp140 protein forms trimers, but do not resemble the viral Env trimer [5].

**Figure 1:**
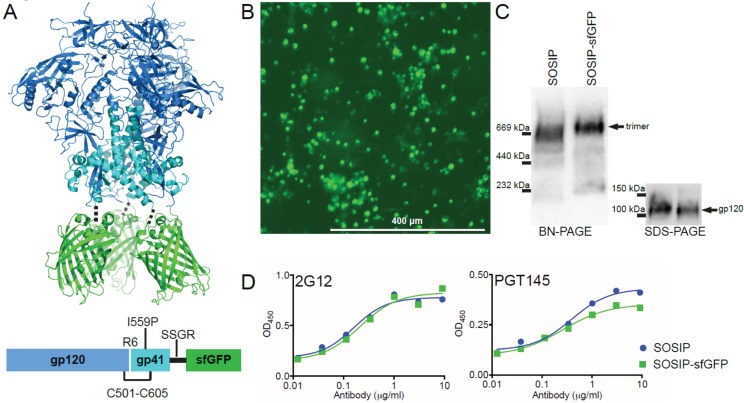
Design and expression of BG505 SOSIP.664-sfGFP (SOSIP-sfGFP). (**A**) **Top:** Model of SOSIP-sfGFP (BG505 SOSIP.664, PDB: 4TVP [13]; sfGFP, PDB: 2B3P [17]). The three gp120 subunits are shown in dark blue, the gp41_ECTO_ subunits in cyan, and sfGFP in green. The three sfGFP subunits were manually fitted to the C-termini of the SOSIP trimer, avoiding potential clashes by visual inspection in PyMol [18]. The dotted line represents the short Ser-Ser-Gly-Arg (SSGR) linker connecting the C-termini of SOSIP to sfGFP. **Below:** 2D schematic of SOSIP-sfGFP with the intersubunit disulfide bond (C501-C605 (“SOS”) [3]); the Ile to Pro substitution at position 559 (I559P (“IP”) [7]); the improved furin cleavage site (hexa-Arg (“R6” [8]) and the SSGR-linker indicated; (**B**) HEK293T cells imaged three days after SOSIP-sfGFP transfection using a fluorescence microscope (EVOS FL (Life Technologies, Bleiswijk, The Netherlands) with an AMEP-4623 objective: Ex/Em: 470/525 nm; magnification: 10x; numerical aperture: 0.3); (**C**) BN-PAGE (**left**) and reducing SDS-PAGE (**right**) analyses performed on HEK293T cell supernatants three days after transfection with a SOSIP or SOSIP-sfGFP encoding plasmid. Both gels were subjected to Western blotting using monoclonal antibodies 2G12 (BN-PAGE) and ARP3119 (SDS-PAGE) to detect Env protein; (**D**) Binding of SOSIP and SOSIP-sfGFP proteins in HEK293T cell supernatant to bNAbs 2G12 and PGT145 as assessed by ELISA. SOSIP and SOSIP-sfGFP proteins were captured onto the solid phase by bNAb 2G12 and probed with biotinylated 2G12 or PGT145.

Using an alternative approach, a soluble native-like HIV-1 Env mimic (BG505 SOSIP.664) was engineered that displays almost all properties of a viral Env spike [6]. The BG505 protein sequence was derived from a clade A virus strain and the protein was stabilized by introducing several mutations (dubbed “SOSIP.664”) [3,6,7,8,9] (Figure 1A). The resulting BG505 SOSIP.664 trimer was highly thermostable and its antigenic profile correlated well to that of Env on the parental virus strain [6]. It was also the first HIV-1 Env trimer for which the high-resolution structure was solved, enabling structure-based vaccine design [10,11,12,13]. Furthermore, immunization studies have shown that BG505 SOSIP.664 induces potent neutralizing antibodies against the neutralization-resistant (tier 2) autologous virus, arguably an important step towards the development of Env immunogens that can induce broad neutralization [14].

A fluorescent soluble native-like Env trimer would be a useful tool for various purposes, e.g., to follow trafficking of the Env using live-cell imaging, to isolate B cells by fluorescence-activated cell sorting (FACS) for the identification of new bNAbs from HIV-1 infected patients, or to track Env antigens in immunized animals. So far, fluorescently labeled gp120 monomers or membrane-anchored Env trimers have been described [15,16]. Here, we report the engineering and extensive characterization of a fluorescent soluble native-like Env trimer composed of BG505 SOSIP.664 fused to superfolder green fluorescent protein (sfGFP).

## 2. Results

### 2.1. Design of a Fluorescent Env Trimer

The BG505 SOSIP.664 trimer (termed SOSIP from hereon) design is compatible with the C-terminal fusion of small tags, such as a His-tag, small epitope tags and AviTag for direct biotinylation [6,19]. Fluorescent proteins (FPs) are significantly larger and thus might affect the folding and/or conformation of the fusion protein [20]. Indeed, we have previously encountered problems with gp140-fusion constructs including reduced precursor cleavage [21,22]. We selected superfolder GFP (sfGFP) for tagging BG505 SOSIP.664 (termed SOSIP-sfGFP), since sfGFP was specifically engineered to retain its fluorescent properties, even when fused to poorly folding proteins [17]. We first verified whether the three sfGFP molecules would clash when attached to the C-terminus of the SOSIP trimer *in silico* (Figure 1A, top). The C-termini of the three gp41_ECTO_ subunits appeared to be sufficiently spaced to accommodate the sfGFP molecules at the base of the trimer (Figure 1A, top). Therefore, we inserted only a short linker (Ser-Ser-Gly-Arg) between the SOSIP and sfGFP moieties (Figure 1A, bottom).

Human HEK293T cells were transiently transfected with a DNA plasmid encoding SOSIP-sfGFP. The transfected cells displayed bright fluorescence, indicating that the C-terminal sfGFP component of the fusion protein was efficiently synthesized and folded (Figure 1B). The supernatant of the cells containing secreted fusion proteins was harvested and analyzed by SDS- and Blue Native (BN)-PAGE, followed by Western Blotting (Figure 1C). Fusion of SOSIP to sfGFP did not affect Env protein expression, cleavage, or trimerization (Figure 1C). Both SOSIP and SOSIP-sfGFP containing supernatants showed similar reactivity to PGT145, an Ab that is highly specific for a quaternary epitope that is only present on native-like Env trimers [23] (Figure 1D). Together, these initial results showed that SOSIP-sfGFP was efficiently expressed in a mammalian cell line, retaining both the fluorescent property of the sfGFP moiety and the native-like conformation of BG505 SOSIP.664. This encouraged us to purify and characterize SOSIP-sfGFP in more detail.

### 2.2. Purification and Characterization of Fluorescent Env Trimers

**Figure 2:**
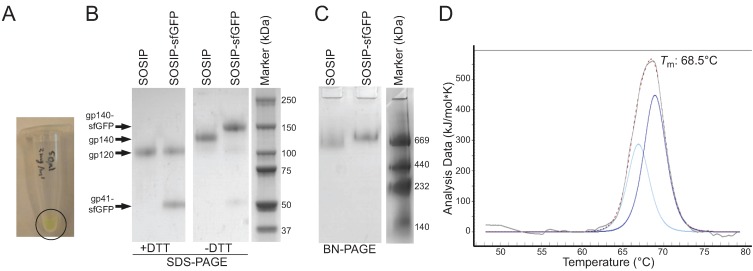
Purification and biochemical characterization of SOSIP-sfGFP. (**A**) Image of an Eppendorf tube containing PGT145-purified SOSIP-sfGFP (2 mg/mL); (**B**) Coomassie-stained reducing and non-reducing SDS-PAGE gels showing SOSIP and SOSIP-sfGFP, both purified with a PGT145-affinity column; (**C**) Coomassie-stained BN-PAGE gel of the same samples shown in B; (**D**) Melting curve of SOSIP-sfGFP obtained by DSC. The raw data is depicted in grey, while the fitted curve, from which the *T*_m_ was derived, is depicted with a dotted line. Deconvolution of the unfolding profile using a non-two state model revealed two peaks, shown in light and dark blue.

Suspension HEK293F cells were transfected with the plasmid encoding SOSIP-sfGFP. We purified the fusion protein using a PGT145 affinity column to select for well-folded SOSIP-sfGFP trimers [24]. The amount of SOSIP-sfGFP obtained from the HEK293F cell supernatant (3.5 mg/L) was similar to the yield of SOSIP (3–4 mg/L) [25] and the protein was green (Figure 2A). Coomassie-stained SDS-PAGE gel analysis revealed that the purity of the preparations was high (>95%) (Figure 2B). The reducing SDS-PAGE gels showed two bands at the expected molecular weights for gp120 and gp41_ECTO_-sfGFP, confirming that SOSIP-sfGFP was fully cleaved (Figure 2B). The non-reducing gel showed only one band at the expected weight for gp140-sfGFP, indicating that spontaneous aggregation by formation of aberrant disulfide bonds in SOSIP-sfGFP did not occur (Figure 2B) [26]. Since SOSIP-sfGFP was purified with PGT145, the SOSIP-sfGFP population consisted solely of trimers as shown in the BN-PAGE (Figure 2C). As expected, SOSIP-sfGFP trimers migrated slower than the SOSIP trimers due to the presence of the sfGFP moieties (Figure 2C). The thermostability of SOSIP-sfGFP trimers, as determined by differential scanning calorimetry (DSC), was slightly increased compared to that of PGT145-purified BG505 SOSIP.664 trimers (Figure 2D) with a midpoint of thermal denaturation (*T*_m_) of 68.5 °C *vs.* 66.7 °C for the wild-type protein [25]. No separate sfGFP melting event was observed, probably because sfGFP has a *T*_m_ beyond the tested temperature range (>80 °C) (Figure 2D) [27].

### 2.3. Morphology and Glycan Composition of Fluorescent Env Trimers

**Figure 3:**
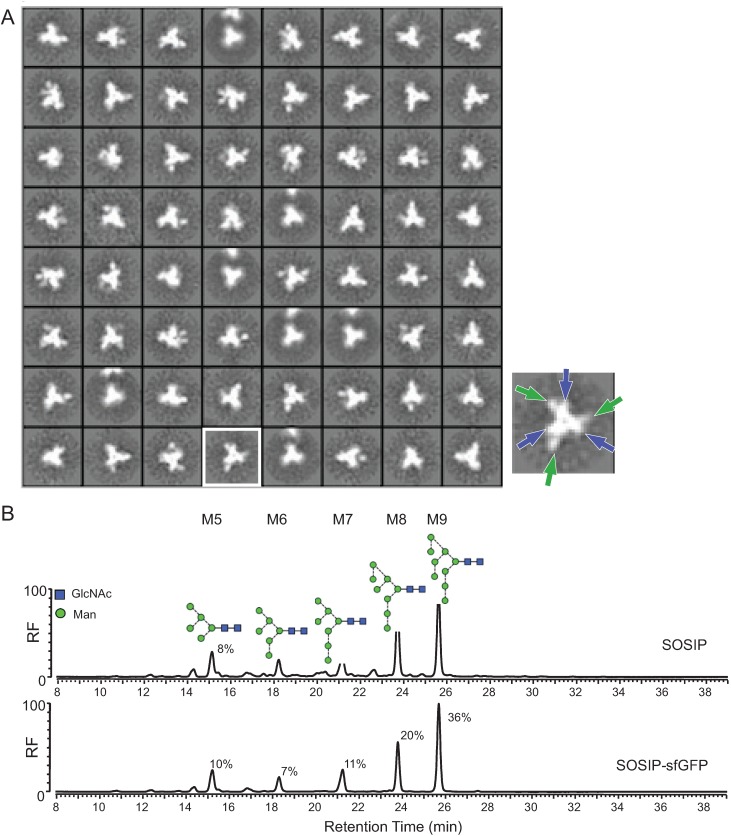
Conformation and glycosylation of SOSIP-sfGFP. (**A**) Negative stain electron microscopy (NS-EM) 2D class averages of PGT145-purified SOSIP-sfGFP. The three propeller blades of the Env trimer (blue arrows) and the protruding densities (green arrows) are highlighted in the image on the right; (**B**) HILIC-UPLC spectra of *N*-linked glycans derived from SOSIP or SOSIP-sfGFP. Peaks corresponding to oligomannose glycans (Man_5-9_GlcNac_2_) are labeled M5-M9. The relative abundance of M5-M9 is given. The smaller peaks that are not highlighted correspond to complex or hybrid-type glycans.

To verify that the individual SOSIP-sfGFP molecules resembled native-like trimers, we analyzed them by negative stain electron microscopy (NS-EM) [6]. Single particles were picked automatically and the resulting reference free 2D class averages showed that practically all SOSIP-sfGFP particles (>95%) formed propeller-blade structures typical for soluble native-like Env trimers [6,24] (Figure 3A). In most of the 2D class averages, protruding densities were visible that can be attributed to the sfGFP moieties (Figure 3A, right picture), although we note that some of these densities could also be ascribed to a slightly more “open” configuration of the Env trimer [24].

Approximately half of the mass of the ectodomains of the HIV-1 Env trimer is made up of *N*-linked glycans that are essential for the folding, conformation, and antigenic structure of Env [28]. The viral Env trimer contains mostly unprocessed oligomannose [29,30,31] and so does the SOSIP trimer, while soluble uncleaved non-native like gp140 trimers or gp120 monomers contain considerably more processed complex glycans [30,31,32]. Misfolded Env trimers on the other hand contain more complex glycans, because of increased accessibility of mannosidases [32]. We determined the type of glycans that are present on SOSIP-sfGFP with glycan profiling using hydrophilic interaction liquid chromatography-ultra performance liquid chromatography (HILIC-UPLC) (Figure 3B). SOSIP-sfGFP trimer was covered mostly with oligomannose glycans (84% of the total glycan content), more than present on SOSIP (66% of the total glycan content) (Figure 3B) [32]. The decreased amount of complex glycans on SOSIP-sfGFP implies that the C-terminal sfGFP actually decreases accessibility for glycan processing by mannosidases.

### 2.4. Antigenicity of Fluorescent Env Trimers

**Figure 4:**
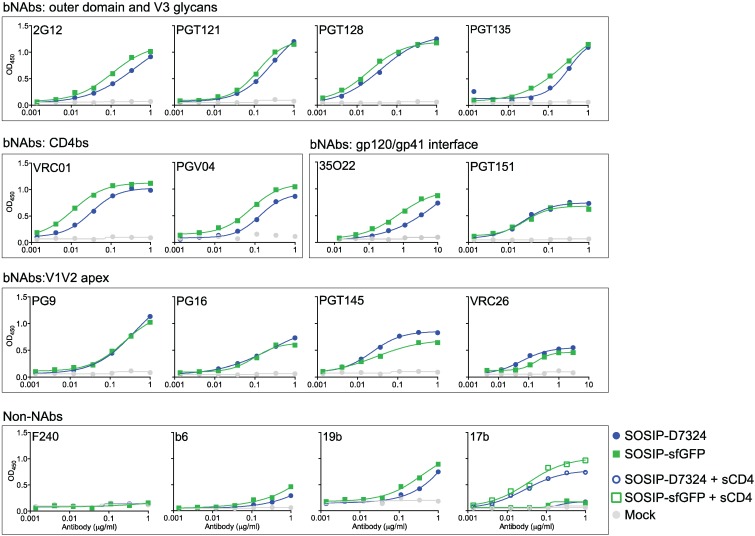
Antigenicity of SOSIP-sfGFP determined by ELISA. Representative ELISA binding curves of PGT145-purified SOSIP and SOSIP-sfGFP to a panel of bNAbs and non-NAbs specific for different epitope clusters on the HIV-1 Env trimer.

We performed enzyme-linked immunosorbent assays (ELISA) by capturing D7324-tagged SOSIP with an anti-D7324 antibody or SOSIP-sfGFP with an anti-GFP antibody. Subsequently, a panel of antibodies was used to probe the antigenic structure of the trimers. Overall, the antigenicity of SOSIP-sfGFP was similar to that of SOSIP (Figure 4). bNAbs against the CD4 binding site (CD4bs VRC01 and PGV04), outer domain and V3 glycans (2G12, PGT121, PGT128 and PGT135) and the gp120/gp41 interface (PGT151 and 35O22) all bound equally well to SOSIP and SOSIP-sfGFP (Figure 4). Most quaternary dependent V1V2-apex bNAbs (PG9, PG16, PGT145, and VRC26) showed slightly decreased reactivity with SOSIP-sfGFP, suggesting that fusion of the relatively bulky sfGFP moiety subtly influenced the conformation of the trimer apex, but this disparity might also be caused by the difference in ELISA capture of SOSIP-sfGFP and SOSIP. Importantly, (poor) binding of non-neutralizing antibodies (non-NAbs) (F240, b6, 19b, 17b) was the same for both trimers (Figure 4). Akin to viral Env spikes, SOSIP trimers can also undergo CD4-induced conformational changes, monitored by increased 17b binding after adding soluble CD4 (sCD4) [6] (Figure 4). Similar results were observed with SOSIP-sfGFP, which shows that the attachment of sfGFP to SOSIP does not impede the CD4-induced conformational changes (Figure 4).

### 2.5. Use of Fluorescent Env Trimers in Flow Cytometry

**Figure 5:**
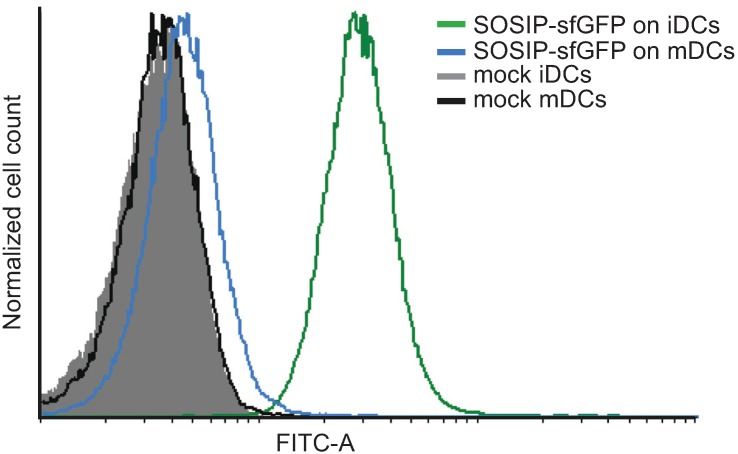
Use of SOSIP-sfGFP in a fluorescence-based assay. FACS histogram plots (FITC channel) of iDCs and mDCs stained with 20 μg/mL SOSIP-sfGFP. No SOSIP-sfGFP to the cells was added as negative control (“mock”).

SOSIP-sfGFP displays all the characteristics of a soluble native-like Env trimer. We determined whether this fluorescent Env trimer is suitable for assays that rely on fluorescence. Immature dendritic cells (iDCs) play an important role in HIV-1 dissemination by capturing the virus via the *N*-linked oligomannose glycans on its Env spike and then transferring the virus to CD4^+^ T cells that subsequently are infected [33]. iDCs were loaded with 20 μg/mL SOSIP-sfGFP and binding was assessed with FACS flow cytometry. A strong fluorescent GFP signal was obtained, confirming that SOSIP-sfGFP was captured by iDCs and could be detected efficiently by flow cytometry (Figure 5) [34]. Mature dendritic cells (mDCs) express lower levels of C-type lectin receptors and therefore captured significantly less SOSIP-sfGFP [35] (Figure 5A).

## 3. Discussion

Recent progress in the development of Env immunogens sparks hope that an HIV-1 Env-based vaccine is possible. The progress includes the induction of neutralizing antibodies against neutralization-resistant primary HIV-1 isolates, the isolation of potent quaternary dependent bNAbs and the elucidation of the atomic structure of the Env trimer, allowing structure-based vaccine design [10,11,12,13,19,36,37,38]. Several of these advances became possible because of the development of the BG505 SOSIP.664 trimer and proteins with similar designs will be good starting points for engineering a new generation of HIV-1 Env-based vaccines. Here, we describe a fluorescent version of BG505 SOSIP.664 that can be exploited for a wide variety of immunological or biochemical experiments. This trimer was easily purified with a simple one-step purification method and preserved all the beneficial properties of BG505 SOSIP.664: efficient trimerization and precursor cleavage; native-like conformation and oligomannose-dominated glycan composition; and efficient binding of bNAbs, including quaternary structure dependent bNAbs, and poor binding of non-nAbs. Furthermore, when used in a fluorescence-based assay, we could readily detect the fluorescent signal coming from the sfGFP molecule.

The robust folding properties of sfGFP make SOSIP-sfGFP very suitable to localize and track SOSIP trimers in living cells [16]. This information is useful for optimizing production efficiency of SOSIP trimers or for comparison of SOSIP processing with that of viral Env. Other possible uses of SOSIP-sfGFP include the isolation of B cells from HIV-1 infected individuals that express potential bNAbs [19] or to study the interaction of cleaved Env trimers with immune cells. SOSIP-sfGFP might also be useful for *in vivo* studies, for example to determine the antigen location in lymphoid organs isolated from vaccinated animals [39].

By introducing point mutations in sfGFP, one could alter the green fluorescent signal into yellow, blue, and cyan fluorescent signal (sfYFP, sfBFP, sfCFP, respectively) [17]. These sfFP could be combined with other native-like trimers, such as those based on the clade B B41 and clade C ZM197M isolates [24,40]. GFP is probably not preferred for tracking antigens in animals using live imaging, because of the high background and limited penetration of the GFP signal. However, a new generation of (near-)infrared fusion FPs such as the recently described infrared fluorescent protein 2 (IFP2) or monomeric near infrared fluorescent protein (iRFP) might be more useful for imaging antigens in living animals, although additional mutations might be necessary to negate aberrant disulfide formation in these FPs or the tendency of iRFP to form dimers [41,42,43]. Based on our results it should then be straightforward to create SOSIP-IFP2 or SOSIP-iRFP proteins.

With this study we have shown that BG505 SOSIP.664 is amenable to attaching medium-sized proteins to its C-terminus, while preserving its native-like conformation. This property can also be exploited for the fusion of native-like trimers to other similarly sized proteins, for example those with immunostimulatory properties [21,22,44], or those with an ability to form nanoparticles allowing for efficient B cell receptor cross-linking and enhancing immunogenicity [45,46].

## 4. Experimental Section

### 4.1. Plasmids

The construction and characterization of sfGFP has been extensively described elsewhere [17]. We cloned the codon optimized nucleotide sequence of superfolder GFP (sfGFP) (Genbank acc. number: AFN88258.1, amino acids 2–238, preceded by Met-Val at amino acid positions −1 and 1) to the 3'-end of the codon optimized nucleotide sequence of BG505 SOSIP.664 [6]. The BG505 protein sequence was derived from a clade A virus strain, BG505, isolated at week six from a pediatric patient that developed broad neutralization within 28 months post-infection [6,47]. The protein was stabilized by several mutations that have been described elsewhere [3,6,7,8,9]. In short, a disulfide bridge (SOS) at positions 501 and 605 was introduced to covalently connect the gp120 and gp41_ECTO_ subunits respectively [3]; an I559P mutation in the gp41_ECTO_ to improve trimer formation [7]; an improved furin cleavage site between gp120 and gp41_ECTO_, by replacing the natural REKR motif by RRRRRR [8]; truncation at position 664 to decrease aggregation [9] and a T332N mutation to enable binding to some glycan-dependent bNAbs [6]. The BG505 SOSIP.664 and sfGFP sequence were separated by a short Ser-Ser-Gly-Arg sequence.

### 4.2. Cells and Protein Production

Adherent HEK293T cells were maintained in Dulbecco’s Modified Eagle’s Medium (DMEM) supplemented with 10% fetal calf serum (FCS), penicillin (100 U/mL), and streptomycin (100 µg/mL). Cells were transfected (~70% confluency) with a plasmid expressing BG505 SOSIP.664 or BG505 SOSIP.664-sfGFP and a plasmid encoding the protease furin in a 1:1 ratio (to ensure cleavage of the gp120 and gp41_ecto_ subunits [8]) using PEImax (Polysciences Europe GmbH, Eppelheim, Germany) as described previously [48]. The supernatant containing SOSIP or SOSIP-sGFP proteins were harvested three days after transfection. HEK293F suspension cells were maintained in FreeStyle medium (Life Technologies). HEK293F cells were transfected at a density of 0.8 × 10^6^–1.2 × 10^6^ with a plasmid expressing BG505 SOSIP.664 or BG505 SOSIP.664-sfGFP and a plasmid encoding the protease furin in a 1:1 ratio using PEImax (Polysciences) as described previously [24]. The supernatant containing SOSIP or SOSIP-sGFP proteins were harvested 6–7 days after transfection, centrifuged and the cleared supernatant was filtered using Steritops (0.22 µm pore size, Millipore, Amsterdam, The Netherlands) before protein purification.

Monocytes, isolated from PBMCs by positive selection with CD14 beads according to MACS miltenyi protocol, were cultured in RPMI 1640 containing 10% FCS and stimulated with 500 U/mL GM-CSF (Schering-Plough, Brussels, Belgium) and 45 ng/mL recombinant IL4 (Biosource, Nivelles, Belgium) on day 0 and 3 and used on day 6 to obtain immature monocyte-derived dendritic cells (iDCs). Mature monocyte-derived DCs (mDCs) were obtained on day 6 after stimulating iDCs on day 5 with 20 μg/mL polyI:C (Sigma-Aldrich Chemie, Zwijndrecht, The Netherlands).

### 4.3. SDS-PAGE, BN-PAGE and Western Blotting

Proteins were subjected to sodium dodecyl sulfate-polyacrylamide gel electrophoresis (SDS-PAGE) and blue native PAGE (BN-PAGE). Purified proteins subjected to PAGE analyses were visualized with Coomassie Blue staining (Bio-Rad, Veenendaal, The Netherlands), while unpurified proteins present in HEK293T supernatants were subjected to PAGE and visualized by Western Blotting using anti-gp120 mAb ARP3119 (0.2 µg/mL) for SDS-PAGE and 2G12 (0.1 µg/mL) for BN-PAGE. Blotted proteins were detected using a horseradish peroxidase (HRP) labeled goat anti-mouse Ab (for ARP3119 mAb) or goat anti-human Ab (for 2G12 mAb).

### 4.4. Protein Purification

BG505 SOSIP.664 and BG505 SOSIP.664-sfGFP trimer proteins were purified essentially as described before [24]. In short, filtered HEK293F cell supernatant was flowed (0.5–1.0 mL/min) over a PGT145 affinity column (prepared as previously described [24]) at 4 °C. The column was washed with three column volumes of 0.5M NaCl and 20mM TrisHCl, pH 8.0. Protein was eluted with 3M MgCl_2_, pH 7.5 and immediately buffer exchanged into TN75 buffer (75 mM NaCl and 20 mM Tris HCl, pH 8.0) using a 100 kDa cutoff Vivaspin20 filter (Sartorius, Goettingen, Germany). Protein concentrations were determined with Nanodrop (Thermo Scientific, Wilmington, DE, USA) using theoretical molecular weight and extinction coefficient and multiplying the molecular weight of the SOSIP.664 moiety with the glycan occupancy factor (×1.82).

### 4.5. Differential Scanning Calorimetry (DSC)

Thermal denaturation was studied using a nano-DSC calorimeter (TA instruments, Etten-Leur, The Netherlands). All SOSIP-sfGFP was first extensively dialyzed against PBS, and the protein concentration then adjusted to 0.25 mg/mL. After loading the sample into the cell, thermal denaturation was probed at a scan rate of 60 °C/h. Buffer correction, normalization, and baseline subtraction procedures were applied before the data were analyzed using NanoAnalyze Software v.3.3.0 (TA Instruments). The data were fitted using a non-two-state model, as the asymmetry of some of the peaks suggested that unfolding intermediates were present.

### 4.6. Enzyme-Linked Immunsorbant Assay (ELISA)

To capture non-pure SOSIP or SOSIP-sfGFP proteins present in 293T supernatants, 96-well half-area plates were coated with bNAb 2G12 (Polymun Scientifc, Klosterneuburg, Austria). The captured proteins were then probed using serially diluted biotinylated 2G12 or PGT145 (kindly provided by Ronald Derking) [49]. Biotinylated 2G12 or PGT145 bNAbs were detected using horseradish peroxidase (HRP)-labeled streptavidin (Sanquin, Amsterdam, The Netherlands). To compare PGT145-purified D7324-tagged BG505 SOSIP.664 trimers and BG505 SOSIP.664-sfGFP trimers, 96-well half-area plates were coated with 10 µg/mL D7324 antibody or 1.25 µg/mL polyclonal anti-GFP antibody (Genscript, Piscataway, NJ, USA). 3 µg/mL of trimer proteins were subsequently captured on the 96-well ELISA plate and tested for antibody binding. To evaluate 17b binding after CD4 attachment, we added soluble CD4 at a fixed concentration of 1.0 µg/mL in the same wells. Antibodies were detected with goat-anti-human (GaH) HRP-labeled antibody (Jackson Immunoresearch, West Grove, PA, USA) in a 1:5000 dilution. OD values from the GaH antibody, which showed some reactivity to the rabbit anti-GFP capture antibody, were subtracted from the obtained OD values. Under these conditions, the signal obtained with 2G12 as the detection Ab, was similar for D7324-tagged SOSIP and SOSIP-sfGFP (Figure 4).

### 4.7. Electron Microscopy

Sample preparation for negative stain electron microscopy (NS-EM) has been described elsewhere [24]. Briefly, PGT145-purified SOSIP-sfGFP was diluted to ~0.03 mg/mL in 50 mM Tris-HCl pH 7.4 and 150 mM NaCl before being absorbed onto carbon-coated copper mesh grids for about 10 s. Contrast was achieved by staining with 2% w/v uranyl formate for 45 s. Data collection and processing methods were based upon those described in [24].

### 4.8. Glycan Profiling

Glycan profiling was done exactly as described elsewhere [32]. In short, Env trimers were resolved by SDS-PAGE. Bands corresponding to gp140 were excised and the *N*-linked glycans attached to gp140 protein were released by *N*-glycosidase F (PNGase F; NEB). Glycans were labelled with 2-aminobenzoic acid (2-AA) [50]. Fluorescently labelled glycans were resolved by hydrophilic interaction liquid chromatography-ultra performance liquid chromatography (HILIC-UPLC) using a 2.1 mm × 10 mm Acquity BEH Amide Column (1.7 μm particle size) (Waters, Elstree, UK) as described before [32]. Fluorescence was measured using an excitation wavelength of 250 nm and a detection wavelength of 428 nm. Data processing was performed using Empower 3 software (Waters Corporation, Milford, MA, USA). The percentage abundance of oligomannose-type glycans was calculated by integration of the relevant peak areas before and after Endoglycosidase H digestion, following normalization.

### 4.9. Flow Cytometry

iDCs (1.0 × 10^5^) were incubated with or without 20 μg SOSIP-sfGFP for 2 h at 37 °C. Unbound SOSIP-sfGFP was removed by washing the cells three times with PBS. Cells were resuspended in FACS buffer (2% FCS in PBS) and analyzed by FACS flow cytometry. Mean fluorescent intensity (MFI) plots were prepared with Cyflogic v. 1.2.1 software (CyFlo, Turku, Finland).

## 5. Conclusions

Here, we have described a soluble HIV-1 Env trimer consisting of the BG505 SOSIP.664 protein trimer fused to three sfGFP moieties. This recombinant fluorescent protein largely retained the antigenic profile, thermostability, glycan composition and overall morphology of the native-like BG505 SOSIP.664 trimer. We conclude that the BG505 SOSIP.664-sfGFP trimer could be a valuable tool in different fluorescence-based assays that require native-like Env trimers.
